# Emerging roles of mesenchymal stem cell-derived exosomes in gastrointestinal cancers

**DOI:** 10.3389/fbioe.2022.1019459

**Published:** 2022-10-20

**Authors:** Naijian Wang, Bing Pei, Xinyi Yuan, Chengxue Yi, Dickson Kofi Wiredu Ocansey, Hua Qian, Fei Mao

**Affiliations:** ^1^ Affiliated Hospital of Jiangsu University, Jiangsu University, Zhenjiang, Jiangsu, China; ^2^ Key Laboratory of Medical Science and Laboratory Medicine of Jiangsu Province, School of Medicine, Jiangsu University, Zhenjiang, Jiangsu, China; ^3^ Department of Clinical Laboratory, The Affiliated Suqian First People’s Hospital of Nanjing Medical University, Suqian, Jiangsu, China; ^4^ School of Medical Technology, Zhenjiang College, Zhenjiang, Jiangsu, China; ^5^ Directorate of University Health Services, University of Cape Coast, Cape Coast, Ghana

**Keywords:** mesenchymal stem cell, exosome, gastrointestinal cancer, drug carrier, miRNA

## Abstract

Gastrointestinal tumours are the most common solid tumours, with a poor prognosis and remain a major challenge in cancer treatment. Mesenchymal stem cells (MSC) are multipotent stromal cells with the potential to differentiate into multiple cell types. Several studies have shown that MSC-derived exosomes have become essential regulators of intercellular communication in a variety of physiological and pathological processes. Notably, MSC-derived exosomes support or inhibit tumour progression in different cancers through the delivery of proteins, RNA, DNA, and bioactive lipids. Herein, we summarise current advances in MSC-derived exosomes in cancer research, with particular reference to their role in gastrointestinal tumour development. MSC-derived exosomes are expected to be a novel potential strategy for the treatment of gastrointestinal cancers.

## Introduction

Gastrointestinal (GI) cancers are malignant tumours of the gastrointestinal tract and the digestive appendages, including the liver and colorectum. According to the World Health Organisation, over 15% of new diagnoses and 17% of cancer deaths are attributed to GI cancers. Among these, the most common GI cancers are colorectal cancer with over 1.8 million cases per year, stomach cancer with 1.03 million cases, and liver cancer with 782,000 deaths (Ren and Xu, 2020). Overall, surgical resection remains the main clinical treatment option for early treatment of colorectal, gastric, oesophageal, and other gastrointestinal cancers. Despite the emergence of new treatments such as neoadjuvant radiotherapy, bacterial therapy, and targeted immunotherapy, their application has not yet been promoted and a significant number of patients still develop distant metastases and drug resistance (Smyth and Moehler, 2019; Soleimanpour et al., 2020; Wang et al., 2021a). Thus, there is an urgent need for a new treatment strategy to improve the outcome and clinical prognosis of patients with GI cancers.

Mesenchymal stem cells (MSCs) are an important source of stem cell therapy in regenerative medicine. Also known as “mesenchymal stromal cells”, MSCs are an important component of the tumour microenvironment (TME) (Whiteside, 2018). MSCs are a population of adult pluripotent cells capable of self-renewal and differentiation into osteoblasts, chondrocytes, and adipocytes, and may differentiate at tumour sites. It is due to their powerful immunomodulatory and immunosuppressive properties, as well as tissue regeneration and repair capabilities that MSC therapy has emerged as a novel clinical treatment strategy for cancer and a variety of diseases with excessive immune responses such as inflammatory bowel disease (IBD) and colorectal cancer (Kang et al., 2018). MSCs play a double-edged sword-like role in tumourigenesis and progression (Liang et al., 2021a). On the one hand, they provide a framework for anchoring tumour cells in the form of tumour stroma and secrete factors that facilitate tumour growth. On the other hand, MSCs present in TME can be transdifferentiated into M2 macrophages or myeloid-derived suppressor cells (MDSC) under the influence of cytokines or chemokines (Ridge et al., 2017).

**FIGURE 1:**
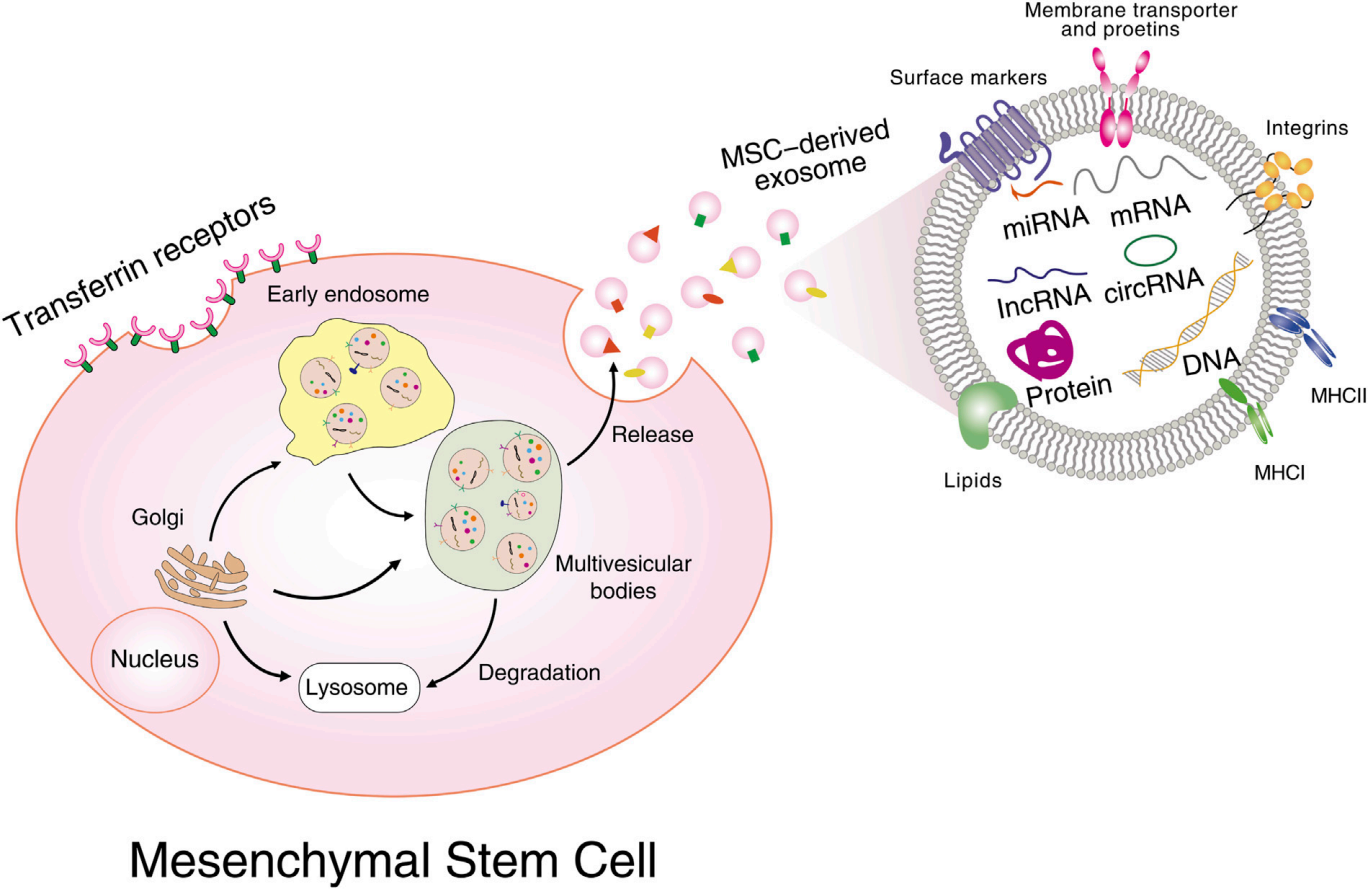
Biogenesis, secretion, and molecular composition of MSC-derived exosomes. Multiple proteins are internalized from the cell surface or transported from the Golgi; nucleic acids are endocytosed and delivered into the endosomes, followed by the formation of intracellular multivesicular bodies (MVB). Further invagination of late endosomal membranes ultimately results in the secretion of exosomes. MVBs are then either taken up by lysosomes for degradation or fused with plasma membrane for releasing all their cargos into extracellular spaces. Exosomal cargoes include proteins, mRNAs, miRNAs, lncRNAs, circular RNAs, DNAs, *etc.*

All types of MSCs have a positive effect on the treatment of cancer and can be a widely promoted therapeutic strategy in clinical practice. The mechanisms of action of MSCs are based on intercellular interactions and paracrine activity. Concerning MSC-derived exosomes, which are considered to be a paracrine interaction and play a powerful immunomodulatory role in a variety of diseases including cancer, the therapeutic functions of MSCs are largely dependent on these derived exosomes (Jafari et al., 2019). Exosomes are bilayer membrane structures containing proteins, lipids, RNA, metabolites, growth factors, and cytokines, and are multifunctional transporters between cells. Figure 1 illustrates the biogenesis, secretion, and molecular composition of MSC-derived exosomes. It has been established by studies that exosomes can transfer biomolecules between tumour cells, stromal cells, fibroblasts, endothelial cells, and immune cells, and act as paracrine mediators to facilitate communication throughout the TME. Thus, exosomes are closely linked to cancer pathogenesis, progression, metastasis, and immune regulation.

In recent years, the role of cancer tissue-derived exosomes in the development and progression of gastrointestinal cancers has been extensively studied (Table 1). Similarly, MSC-derived exosomes have also been shown to be involved in the development of various gastrointestinal cancers such as gastric cancer, colorectal cancer, hepatocellular cancer, and pancreatic cancer. Unfortunately, a systematic review on MSC-derived exosomes in this context is still relatively lacking, and the review written by Zhao et al. (Zhao et al., 2021) is a way forward to filling this gap. In their review, they clearly highlight the anti-tumour and pro-tumour effects of MSC-derived exosomes on GI cancers and summarise the reasons for the different roles played by exosomes in the development of GI cancers. As a constantly expanding field with great potential, research on the use of MSC-derived exosomes on GI cancers needs regular assessment of the progress made. Thus, in this review, we present a more detailed overview of the role of MSC-derived exosomes in GI cancers and discuss their potential clinical implications and future research directions. This will contribute to driving the field in understanding the current status of progress, identifying gaps, and addressing challenges towards clinical application.

**TABLE 1:** Effects of cancer tissue-derived exosomes on gastrointestinal cancers.

Cancer types	Exosomal cargo	Studymodel	Function	Mechanism	Reference
Pancreatic cancer	miR-19a	*In vitro* and *in vivo*	Involve in the pathogenesis of pancreatic cancer-associated diabetes	Induce β-cell dysfunction by targeting ADCY1 and EPAC2	Pang et al. (2021)
	miR-30b-5p	*In vitro* and *in vivo*	A potential diagnostic marker for PDAC	Promote tumour angiogenesis through the inhibition of GJA1 expression	Chen et al. (2022)
	Myoferlin	*In vitro*	Influence the ability of human endothelial cells to transfer nucleic acids	Promote tumour growth and angiogenesis through the regulation of VEFG	Blomme et al. (2016)
	ZIP4	*In vitro* and *in vivo*	A novel diagnostic biomarker for pancreatic cancer	Stimulate proliferation, migration, and invasion of non-metastatic pancreatic cancer cells	Jin et al. (2018)
Colorectal cancer	circPACRGL	*In vitro* and *in vivo*	Play an oncogenic role in CRC proliferation and metastasis	Promote progression of colorectal cancer *via* the miR-142-3p/miR-506-3p-TGF-β1 axis	Shang et al. (2020)
	HSPC111	*In vitro* and *in vivo*	A potential therapeutic target for preventing CRC liver metastases	Promote colorectal cancer liver metastasis by reprogramming tumour-associated fibroblast lipid metabolism	Zhang et al. (2022)
	miR-106b-3p	*In vitro* and *in vivo*	A potential prognostic biomarker and therapeutic target for CRC	Promote metastasis by downregulating the expression of DLC-1	Liu et al. (2020a)
	Wnt4	*In vitro*	Enhance β-catenin translocation to the nucleus in CRC cells	Activate the Wnt/β-catenin pathway to induce migration and invasion	Huang et al. (2018)
	CAPS1	*In vitro*	Promote epithelial cell migration	Overexpression of CAPS1 could alter the expression pattern of exosomal proteins involved in cell migration	Wu et al. (2019)
Gastric cancer	miR-301a-3p	*In vitro* and *in vivo*	A promising predictors and potential therapeutic target for GC metastasis	Promote gastric cancer malignant behavior and metastasis through HIF-1α accumulation	Xia et al. (2020)
	miR-130a	*In vitro* and *in vivo*	A potential biomarker for monitoring the activity of GC	Target vascular endothelial cells C-MYB to activate gastric cancer angiogenesis	Yang et al. (2018)
	X26nt	*In vitro* and *in vivo*	Promote the proliferation, migration, and tube formation of human umbilical vein endothelial cells	Increase angiogenesis and vascular permeability by targeting VE-cadherin	Chen et al. (2021)
Liver cancer	miR-4454	*In vitro*	Promote the proliferation, migration,invasion, and vascularization and accelerate cycle arrest, apoptosis, and ROS of HepG2 cells	Promote Hepatic Carcinoma Progression by Targeting Vps4A and Rab27A	Lin et al. (2021)

ADCY1, Adenylate cyclase 1; DLC-1, Deleted in Liver Cancer-1; EPAC2, exchange protein activated by cyclic-AMP 2; GC, gastric cancer; GJA1, Gap Junction Protein Alpha 1; HIF, hypoxia-inducible factors; PDAC, pancreatic ductal adenocarcinoma; TGF-β1, transforming growth factor-β1; VE-cadherin, vascular endothelial cadherin; VEFG, vascular endothelial growth factor; Vps4A, vacuolar protein sorting 4.

## The dual role of MSCs in cancers

MSCs are adult stem cells with high differentiation potential and self-renewal ability (Wei et al., 2013). MSCs are found in a wide variety of tissues, initially isolated from bone marrow and later from adipose tissue, muscle, peripheral blood, hair follicles, teeth, placenta, and umbilical cords (Hmadcha et al., 2020). MSCs can differentiate into different cell types such as osteoblasts, chondrocytes, myoblasts, and adipocytes, giving rise to a variety of mesenchymal tissues. Interactions are initiated by cytokines, chemokines, extracellular vesicles, inflammatory stimuli, or co-culture with other cells (Song et al., 2020). Due to their immunomodulatory and cell survival-promoting functions, as well as their ease of access and efficient proliferation *in vitro*, MSCs have been widely studied and researched since their discovery, and a range of MSC-based cell therapies are now available in the clinic. In its application in inflammation, infection, metabolic abnormalities, immune disorders, tissue damage, and many other diseases, regenerative medicine solutions using MSCs as a therapeutic tool has shown significant effects (Yuan et al., 2021).

Overall, MSCs have a dual role in promoting and inhibiting tumourigenesis and development. In addition to protecting the host from foreign invasion, the body’s immune system recognises tumour antigens and destroys malignant tumours. Thus, tumour growth, invasion and metastasis are important aspects of the tumour immune escape mechanism. MSCs have powerful immunosuppressive functions that support tumour cell evasion from immune surveillance. In the tumour microenvironment, MSCs are activated mainly by the pro-inflammatory factors tumournecrosis factor-α (TNF-α), Interferon-γ (IFN-γ) or interleukin-1β (IL-1β) and interact with different types of immune cells such as B cells, T cells, dendritic cells, NK cells, and macrophages by secreting mediators and soluble factors such as indoleamine 2,3-dioxygenase (IDO), prostaglandin E2 (PGE2), and NO (Rhee et al., 2015). MSCs play an important role in promoting tumour angiogenesis through the release of high levels of cytokines and pro-angiogenic growth factors, including vascular endothelial growth factor (VEGF), fibroblast growth factor-2 (FGF-2), IL-8, IL-6, angiopoietin, and transforming growth factor-β (TGF-β) (Feng and Chen, 2009; Du et al., 2016). Cancer-associated fibroblasts (CAFs) are present in most aggressive tumours and secrete stromal cell-derived factor-1 (SDF-1), which promotes angiogenesis and tumour growth (Orimo et al., 2005). It has been shown that MSCs are resting fibroblasts from which they can be transformed into CAFs and thus play a pro-tumour role (Kalluri, 2016). In many tumours, molecules such as hepatocyte growth factor (HGF), epidermal growth factor (EGF), platelet-derived growth factors (PDGF), and TGF-β can act as epithelial-mesenchymal transformation (EMT)-inducible signals. Interestingly, these factors are secreted by MSCs and activate a range of EMT-promoting transcription factors to deliver EMT-promoting signals (Tse and Kalluri, 2007). Chen et al. (Chen et al., 2015) report that paracrine factors from adipose MSCs, such as ZEB2, ZEB1, Twist, Slug, and Snail, enhance the metastatic capacity of colon cancer cells in a contact-dependent mode *via* the Wntsignalling pathway in a co-culture model of colon cancer cells. In addition to conferring tumour support by suppressing immune responses in the tumour microenvironment, promoting angiogenesis, transforming to CAFs, and promoting tumour metastasis, MSCs can also mediate inhibition of apoptosis and increase cancer stem cells (CSCs) (Liang et al., 2021a).

In contrast, many other studies have shown that MSCs have a tumour suppressive profile. Recently, the effective role of immunologically activated umbilical cord blood-derived MSC (hucMSC) induced by the TLR7 agonist imiquimod in reducing the viability, proliferation, migration, and invasion of A549 cells and enhancing their apoptosis has been documented (Ye et al., 2021). Notably, the AKT signalling pathway is a central node in many signalling pathways and is closely associated with the survival, invasion, and migration of most tumour cells (Revathidevi and Munirajan, 2019). In Kaposi’s sarcoma model, Khakoo et al (Khakoo et al., 2006) found that intravenously administered MSCs could reach tumour sites and significantly reduce the proliferation of tumour cells. They further found that MSCs could inhibit the activation of AKT protein kinase *in vitro* but not in all tumours and primary cell lines and that their tumour suppressive effect was related to their ability to inhibit AKT activity in target cells *in vivo*. In addition, hucMSC could also inhibit the migration of glioma cells by down-regulating the AKT pathway (Dasari et al., 2010). It is clear from these findings that the regulation of cell signalling is one of the ways in which MSCs exert their anti-tumour effects. Lu et al. (Lu et al., 2008)isolated bone marrow MSCs (BMSCs) from mouse bone marrow and co-cultured them with mouse liver cancer H22, lymphoma (YAC-1 and EL-4), and rat insulinoma INS-1 cells. MSCs were found to inhibit mouse tumours both *in vivo* and *in vitro* by up-regulating the mRNA expression of p21, a negative regulator of the tumour cell cycle, and the expression of caspase 3, an apoptosis-related protease. Moreover, MSCs can also inhibit tumour growth by increasing inflammatory cell infiltration, regulating the cell cycle, and inhibiting angiogenesis in various ways (Chen et al., 2021).

## The multiple effects of MSC-derived exosome on cancers

**FIGURE 2:**
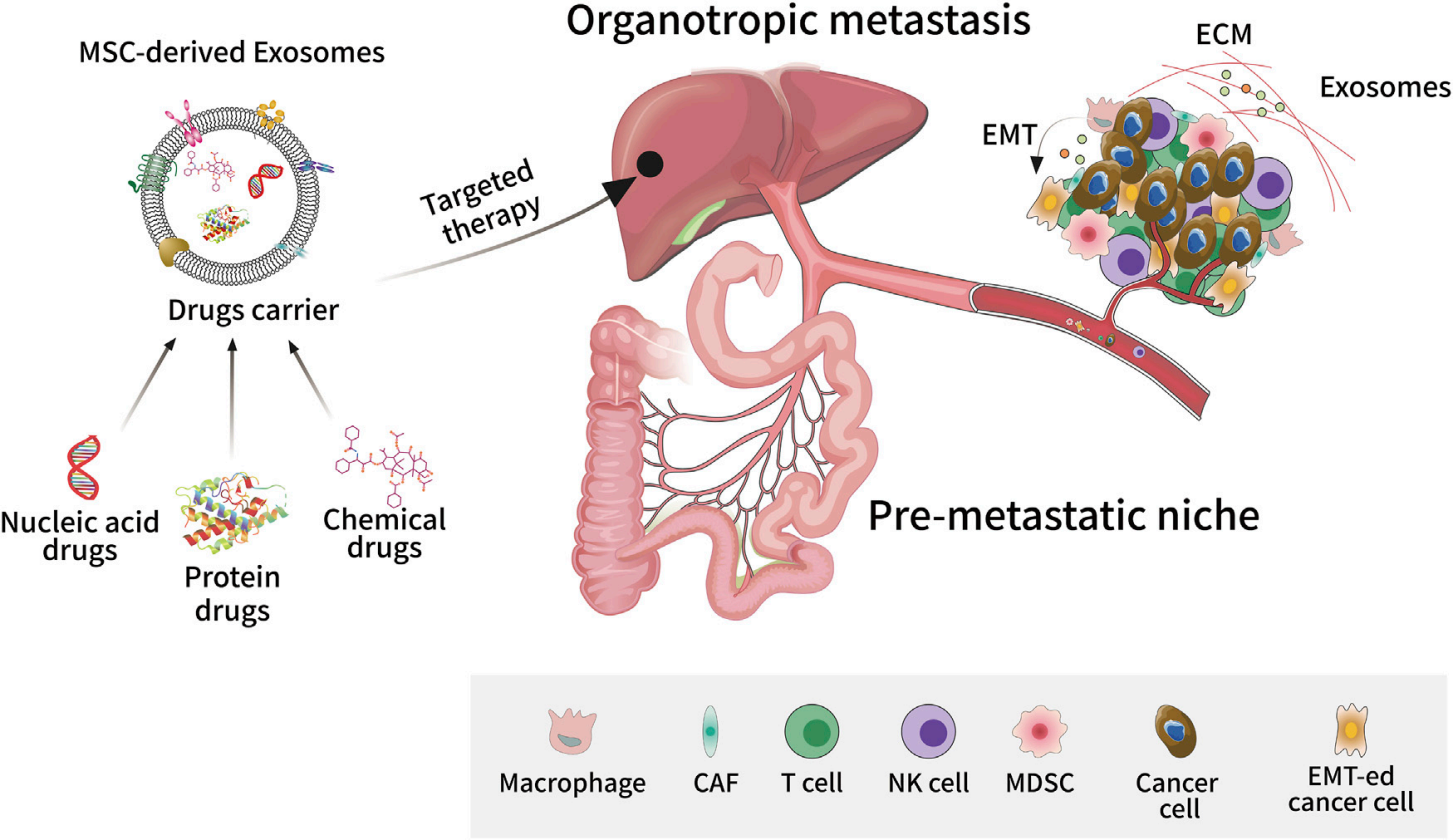
Exosome-targeted therapy in tumour metastasis. As a new type of drug carrier, exosomes can be loaded with different types of compounds such as small molecule chemical drugs, proteins, and nucleic acids to target tumour tissues and exert personalized therapeutic effects. Exosomes play an important role in tumourigenesis and metastasis, promoting epithelial-mesenchymal transition, angiogenesis, and extracellular matrix remodeling in the tumour microenvironment. Exosomes can also promote the formation of pre-metastatic niches by assisting tumour cells to escape immune surveillance, allowing cancer cells to invade and colonize distant organs.

It is generally accepted that MSCs exert their immunomodulatory functions mainly through the paracrine pathway, with their derived exosomes being one of the bioactive substances that have received the most attention. Studies have shown that exosomes are involved in multiple stages of tumourigenesis and development, and as a new type of drug carrier, exosomes are unique in the field of cancer-targeted therapy and personalized therapy (Figure 2). Just like other exosomes, MSC-derived exosomes have also been reported to closely relate tumour proliferation, apoptosis, metastasis, angiogenesis, chemotherapy/radiotherapy resistance, and CSC generation.

### Tumour growth

The effect of exosomes on tumour progression has been widely reported in the last decade or so. MSC-derived exosomes have an impact on tumourigenesis and progression in either a supportive or inhibitory manner, and tumour-associated miRNAs enriched in these exosomes are closely associated with promoting or inhibiting cancer cell proliferation. For example, miR-130b-3p, a potential therapeutic target and biomarker for lung cancer, is upregulated in lung cancer and acts as a promoter of lung cancer (Tian et al., 2016). A study conducted by Guo et al. (Guo et al., 2021) showed that MSC-derived exosomes deliver miR-130b-3p to lung cancer cells and block the NFE2L2/TXNRD1 pathway by inhibiting FOXO3 to achieve the effects of promoting cancer cell proliferation, migration, and invasion while inhibiting cancer cell apoptosis. Similarly, in malignant tumours such as kidney cancer, breast cancer, and nasopharyngeal carcinoma, MSC-derived exosomes can exert oncogenic and pro-carcinogenic effects through specific miRNAs (Du et al., 2014; Zhao et al., 2019; Zhou et al., 2019). Furthermore, BMSC-derived exosomes enhance the expression of VEGF in tumour cells by activating the extracellular signal-regulated kinase 1/2 (ERK1/2) pathway and exerting a pro-cancer effect (Zhu et al., 2012). In addition to higher miRNA levels, other factors such as higher amounts of cytokines and adhesion molecules in patient-derived exosomes may also be involved in tumour promotion.

Contrary to this observation, miRNAs, proteins, and lncRNAs enriched in MSC-derived exosomes also play a role in cancer suppression. miR222-3p is highly expressed in BMSC-derived exosomes (Furuta et al., 2016), which is delivered to negatively regulate the IRF2/INPP4B signaling pathway, thus, inhibiting proliferation and promoting apoptosis in acute myeloid leukemia (AML) cells by targeting IRF2 (Zhang et al., 2020). In addition to BMSC-derived exosomes, hucMSC and adipose MSCs (AMSC)-derived exosomes also have anti-oncogenic properties (Takahara et al., 2016; Maffey et al., 2017). AMSC-derived exosomes inhibit prostate cancer by delivering miR-145 to reduce Bcl-xL activity and promote apoptosis *via* the caspase-3/7 pathway (Takahara et al., 2016).

### Metastasis/invasion

EMT is the cellular process by which cells change from an epithelial phenotype to a mesenchymal phenotype, reducing intercellular adhesion and improving migration. Evidence suggests that the pro-metastatic effects of MSC-derived exosomes in tumour cells are associated with EMT induction. Studies have shown that part of the mechanism by which MSCs induce the invasion of breast cancer cells is *via* exosomes secreted by MSCs (Maffey et al., 2017). Zhou et al. (Du et al., 2016) previously demonstrated that hucMSC-derived exosomes induce EMT *via* the ERK pathway, promoting the invasive and migratory potential of breast cancer cells, and causing malignant tumour progression and metastasis.

### Angiogenesis

MSC-derived exosomes play an intermediary role in intercellular signalling, which includes angiogenic signals, and thus, play a pro- or anti-angiogenic role. To date, no consistent conclusions have been drawn regarding the role of MSC-derived exosomes in angiogenesis in the tumour environment. A recent study found that exosomes isolated from BMSC conditioned media could transfer several angiogenic-promoting miRNAs into HUVEC, thus promoting angiogenesis *in vivo* and playing an important role in stem cell-endothelial cell communication (Gong et al., 2017). Wnts is a potent angiogenic factor and its signalling pathway plays a major role in angiogenesis and vascular remodelling (Olsen et al., 2017). McBride (McBride et al., 2017) and colleagues reported that exosomes isolated from BMSCs could transport Wnt3a to stimulate fibroblast proliferation and enhance angiogenesis *in vitro*. Moreover, exosomes derived from placental MSCs and adipose MSCs have been reported to have pro-angiogenic properties (Salomon et al., 2013; Lopatina et al., 2014).

The anti-vascular remodeling properties of MSC-derived exosomes have also been confirmed by several other studies. For example, miR-16, a miRNA known to target VEGF, is enriched in MSC-derived exosomes. A study found that miR-16 in MSC-derived exosomes is involved in the inhibition of angiogenesis by down-regulating the expression of VEGF and CD31 in breast cancer cells (Lee et al., 2013). Pakravan et al. (Pakravan et al., 2017) similarly found that MSC-derived exosomes inhibit angiogenesis *in vitro* by regulating the mTOR/HIF-1α/VEGF signalling axis in breast cancer cells. The concentration of oncogenic proteins, cytokines, and adhesion molecules may also be associated with exosome-mediated angiogenesis.

### Regulation of the immune response

MSC-derived exosomes have a wide range of immunomodulatory capabilities similar to those of MSCs. Exosomes not only act as natural antigen carriers, but also as presenters to regulate direct and indirect antigen presentation, and stimulate adaptive and innate immune responses. Exosomes also act as carriers for the transfer of antigenic peptides or bioactive molecules, which in turn regulate other immune cell subsets (Thery et al., 2009). BMSC-derived overexpression of IDO-1 exosomes reduces IFN-γ expression in DCs and T cells (He et al., 2018). BMSC-derived exosomes have also been reported to induce immature IL-10-secreting DC activation, increase Foxp3^+^ Treg cell numbers, and suppress inflammatory T helper 17 (Th17) responses (Favaro et al., 2016). Other sources of MSC-derived exosomes are also involved in the regulatory processes of the immune system. AMSC-derived exosomes have been reported to inhibit the proliferation and activation of stimulated T cells, while hucMSC-derived exosomes produce immunosuppressive-related cytokines by binding to monocytes in human peripheral blood mononuclear cells (PBMCs) and producing M2 macrophages (Blazquez et al., 2014; Hu et al., 2020).

## Roles of MSC-derived exosomes in GI cancers

As earlier mentioned, MSC-derived exosomes are closely associated with tumourigenesis and progression. The components of exosomes mainly include proteins, nucleic acids, and lipids, all of which participate in the cancer process. Of these, the role of miRNAs in GI cancer has been most widely studied (Table 2). This section focuses on the role of MSC-derived exosomes and their clinical applications in GIcancer such as gastric, colorectal, hepatocellular, and pancreatic cancers.

**TABLE 2:** The role of MSC-derived exosomal miRNAs in GI cancer.

Cancer types	Exo-miRNA	Study model	Source of exosomes	Function	Mechanism	Reference
Gastric cancer	miR-1228	*In vitro*	hucMSCs	Inhibit GC growth	Inhibit MMP-14 upregulation-induced invasion and migration of gastric cancer cells	Chang et al. (2021)
	miR-301b-3p	*In vitro* and *in vivo*	N/A	Induce drug resistance, proliferation and migration of GC cells	Promote multidrug resistance of gastric cancer cells by inhibiting TXNIP	Zhu et al. (2022)
Colorectal cancer	miR-431-5p	*In vitro* and *in vivo*	hucMSCs	Inhibit CRC growth	Inhibit CRC progression by suppressing PRDX1	Qu et al. (2022)
	miR-16-5p	*In vitro* and *in vivo*	hBM-MSCs	Inhibit CRC cell proliferation, migration and invasion	Stimulates apoptosis in CRC cells by downregulating ITGA2	Xu et al. (2019a)
	miR-22-3p	*In vitro*	hBM-MSCs	Inhibit CRC cell proliferation, migration and invasion	Inhibit CRC cells proliferation and invasion by mediating the RAP2B/PI3K/AKT pathway	Wang and Lin, (2021)
	miR-424	*In vitro* and *in vivo*	hBM-MSCs	Promote CRC cell proliferation and migration	Suppress tumour growth in CRC by upregulating TGFBR	Zhang et al. (2021a)
	miR-30a	*In vitro* and *in vivo*	hCC-MSCs	Promote the tumourigenicity of colon cancer	Promote CRC cells proliferation, migration and metastasis by inhibiting MIA3 expression	Du et al. (2021)
Liver cancer	miR-199a/b-3p	*In vitro* and *in vivo*	AMSCs	Improve the sensitivity of liver cancer cells to chemotherapeutic drugs	Suppresses HCC growth by inhibiting the PAK4/Raf/MEK/ERK pathway	Hou et al. (2011)
	miR -122	*In vitro* and *in vivo*	AMSCs	Improve the antitumour efficacy of chemotherapeutic agents in HCC	Enhance HCC cells sensitivity to chemotherapeutics by altering the expression of miR-122 target genes in HCC cells	Lou et al. (2015)
Pancreatic Cancer	miR-124	*In vitro* and *in vivo*	hBM-MSCs	Inhibit tumour growth	Inhibit the proliferation, invasion, migration, and EMT of pancreatic tumour cells	Xu et al. (2020)
	miR-1231	*In vitro* and *in vivo*	hBM-MSCs	Inhibit the activity of PC	Inhibit PC cell proliferation, migration, invasion and adhesion to the matrix	Shang et al. (2019)
	miR-128-3p	*In vitro*	hucMSCs	Inhibit PC growth	Suppress the proliferation, invasion, and migration of PANC-1 cells	Xie et al. (2022)
	miR-100-5p	*In vitro* and *in vivo*	hucMSCs	Promote the growth of pancreatic ductal adenocarcinoma	Promote Panc-1 and BxPC3 cell growth by increasing proliferation and migration	Ding et al. (2021a)

AMSCs, adipose tissue-derived MSCs; CRC, colorectal cancer; EMT, epithelial-mesenchymal transformation; hBM-MSCs, human bone marrow-derived MSC; HCC, hepatocellular carcinoma; hCC-MSCs, human CRC-derived MSCs; hucMSCs, human umbilical cord-derived MSCs; GC, gastric cancer; MMP, matrixmetalloproteinases; PANC, pancreatic cancer; PC, pancreatic cancer; PRDX1, peroxiredoxin 1.

### Gastric cancer

Gastric cancer (GC) remains a thorny and unsolved clinical challenge. With over one million new cases estimated each year, GC is considered the fifth most diagnosed malignancy in the world (Smyth et al., 2020). In 2018, the global age-standardized gastric cancer incidence and mortality rates were 11.1 and 8.2 per 100,000 people respectively (Thrift and El-Serag, 2020). There are many risk factors for the development of GC, including diet, smoking, alcohol consumption, and *Helicobacter pylori* and Epstein-Barr virus (EBV) infections (Machlowska et al., 2020). Systemic chemotherapy, radiotherapy, surgery, immunotherapy, and targeted therapy are all available as treatment options for GC (Joshi and Badgwell, 2021). In recent years, multidisciplinary treatment has been widely accepted, and more clinicians are opting for triple combination chemotherapy, which can cure GC (Chalabi, 2021). Available evidence has shown that exosomes play an important role in the progression of GC, including tumourigenesis, metastasis, angiogenesis, immune evasion, and drug resistance (Fu et al., 2019). The promise of exosomes, particularly of MSC origin, provides hope and a new perspective on the future of GC prevention and treatment.

It is documented that hBM-MSCs promote GC growth by regulating c-Myc (Chen et al., 2018), which positively regulates the progression of GC. Gu et al. (Gu et al., 2016) found that MSC-derived exosomes could induce EMT and enhance the migration and invasion of GC cells through activation of the AKT pathway, thereby promoting the development and metastasis of GC. Similarly, a study conducted by Jin et al. (Qi et al., 2017)reports that exosomes secreted by hBMSCs have the ability to activate the Hedgehog signalling pathway to promote the proliferation, differentiation, and metastasis of GC cells. These findings provide new evidence for the involvement of MSC-derived exosomes in GC progression. Exosomes may be a novel mediator of the role of MSCs in GC promotion, and MSC-derived exosomes may become a novel therapeutic target for GC treatment.

MSC-derived exosomes are also expected to be efficient nanocarriers to effectively hinder gastric carcinogenesis. Their cargoes like miR-1228 negatively regulate NF-κB activity in GC cells while inhibiting EMT (Jia et al., 2013). Chang et al. (Chang et al., 2021)transfected MSC with lentiviral vectors carrying overexpressed and disrupted sequences of miR-1228 and its downstream target MMP-14, and subsequently extracted exosomes. It was found that BMSC-derived exosomes overexpressing miR-1228 inhibited the growth of GC cells by down-regulating the expression of MMP-14. The same could be demonstrated in another study where L-PGDS served as a potential drug to inhibit the proliferation of GC cells through the PPARγ signalling pathway (Fukuoka et al., 2015). You (You et al., 2022) and others found that MSC-derived extracellular vesicles overexpressing L-PGDS could inhibit GC progression by regulating GC cell stemness and inhibiting STAT3 phosphorylation. This suggests that MSC-derived exosomes could be an effective vehicle for GC therapy.

MSCs play an important role in chemotherapy resistance (Roodhart et al., 2011). There is now evidence that MSCs are associated with microenvironment-mediated drug resistance through the production of multiple factors, circulating macromolecules, and activation of certain signalling cascades to protect tumour cells from chemotherapeutic agents (Houthuijzen et al., 2012; Castells et al., 2013; Kucerova et al., 2013). A growing number of studies also suggest the involvement of MSC-derived exosomes in mediating chemoresistance in GC. Ji et al. (Houthuijzen et al., 2012)first reported that MSC-derived exosomes protect GC cells from chemotherapy-induced apoptosis by upregulating multidrug resistance-associated proteins mainly through their protein activation of the CaM-Ks/Raf/MEK/ERK signalling cascade. Another study (Ji et al., 2015) also showed that miR-301b-3p in exosomes from MSCs promotes multi-drug resistance in GC cells by inhibiting thioredoxin interacting protein (TXNIP). This suggests that MSC-derived exosomes are one of the key regulators of drug resistance in GC cells. Targeting the interaction of MSC-derived exosomes with cancer cells may help to improve the efficacy of GC chemotherapy.

### Colorectal cancer

**FIGURE 3:**
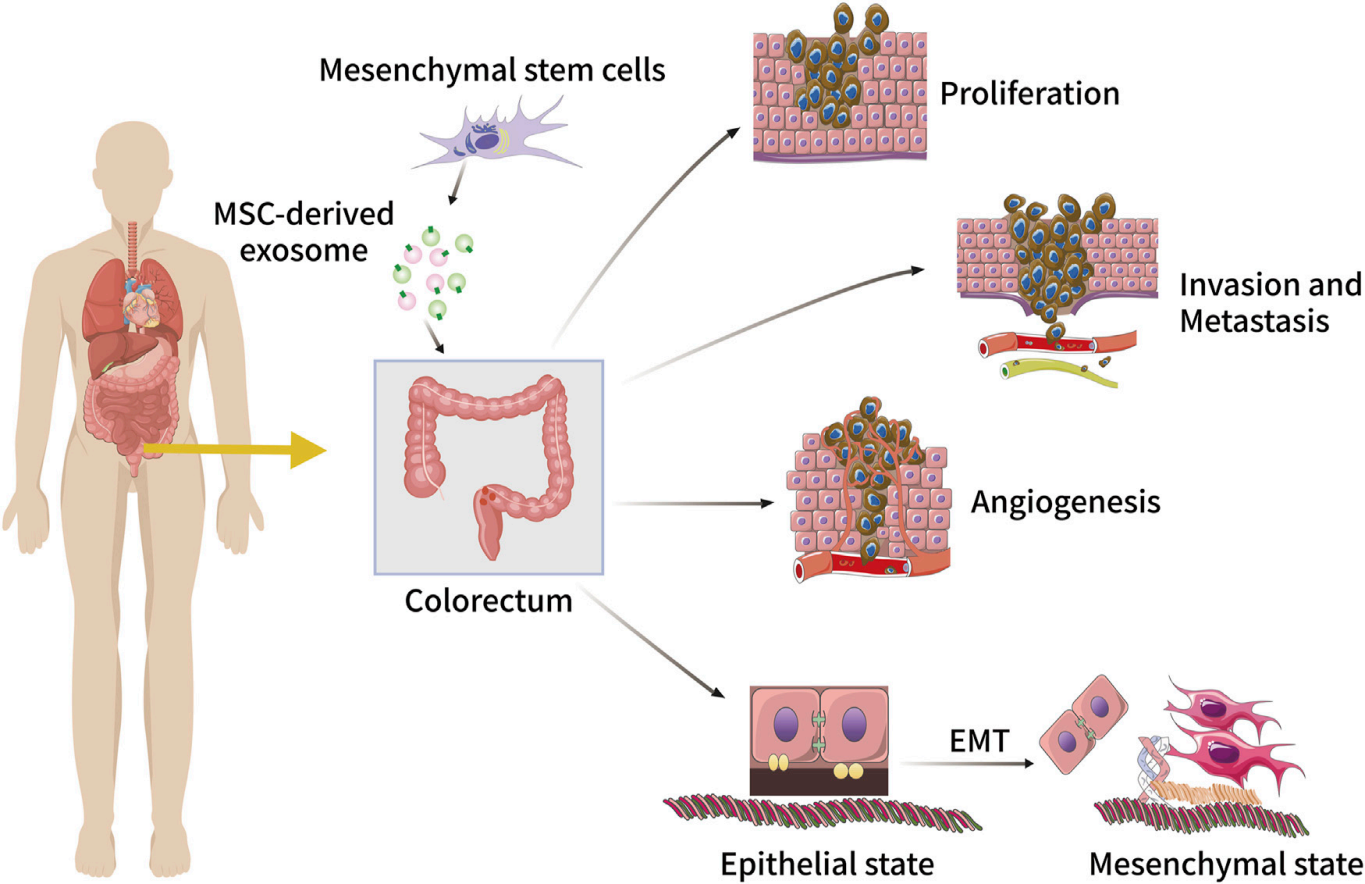
The role of MSC-derived exosomes in CRC initiation and progression, including tumour cell proliferation, invasion, metastasis, angiogenesis, and EMT.

Colorectal cancer (CRC) is the third and second most common cancer in terms of diagnosis and mortality respectively (Kishore and Bhadra, 2021). Globally, 1.8 million cases of CRC were reported in 2018 and 88,100 CRC-related deaths occurred the same year (Bray et al., 2018). Metastatic disease is seen in 20% of patients with newly diagnosed CRC and in a further 25% of patients who develop metastases after presenting with localised lesions (Biller and Schrag, 2021). Conventional treatment options include surgery, chemotherapy, radiotherapy, and immunotherapy (Johdi and Sukor, 2020). Depending on the specific site of the disease and its progression, these treatments can be used in combination (Van Cutsem et al., 2014; Van Cutsem et al., 2016; Yoshino et al., 2018). Epidemiological studies have identified a range of risk factors associated with CRC, including a family history of CRC or associated genetic disorders (e.g. Lynch syndrome, familial adenomatous polyposis), personal medical history (e.g. IBD and diabetes), lifestyle and dietary habits (e.g. smoking, alcohol consumption, consumption of processed meat), and bacterial infections (e.g. *Bacteroides fragilis, Escherichia coli*) (Handel et al., 2020; Kim et al., 2020; McNabb et al., 2020). As mentioned earlier, disease progression, metastasis, and drug resistance in a number of patients with CRC have been associated with exosomal cargo (Cheshomi and Matin, 2018; Kletukhina et al., 2019) (Figure 3).

Just like in other cancers, MSC-derived exosomes have both inhibitory and promotive effects on CRC. A recent study showed that the upregulation of hucMSC-derived miR-431-5p inhibits CRC progression by suppressing PRDX1, with a predictive effect on the prognosis of CRC patients (Qu et al., 2022). The mRNA expression level of ITGA2 has been found to be significantly higher in gastric cancer tissues compared to normal tissues (Chuang et al., 2018), is closely associated with other cancers such as pancreatic cancer and CRC, and therefore could be a potential therapeutic target for these cancers (Sakthianandeswaren et al., 2011; Ferraro et al., 2014; Li et al., 2021a). Moreover, ITGA2 is highly expressed in CRC tissues and is a target gene for miR-16-5p (Xu et al., 2019a). Further studies *in vivo* and *in vitro* reveal that BMSC-derived exosomes overexpressing miR-16-5p inhibit the proliferation, migration, and invasion of CRC cells while stimulating CRC cell apoptosis through the downregulation of ITGA2. Similarly, Wang et al. (Wang and Lin, 2021) reported that miR-22-3p in MSC-derived exosomes also inhibits the development of CRC. miR-22-3p’s downstream target gene is RAP2B, located on 3q25.2, an oncogene that is highly expressed in a variety of tumours and plays an important role in promoting tumour cell proliferation and metastasis (Zhu et al., 2020a; Li et al., 2022a). MSC-derived exosomal miR-22-3p inhibits CRC cell proliferation and invasion by regulating the RAP2B and PI3K/AKT pathways, leading to the inhibition of CRC. Notably, MSC-derived exosomal miRNA-3940-5p can also inhibit CRC metastasis by targeting integrin α6 (Li et al., 2021b). The miR-146a/SUMO1 axis is also a pathway for MSC-derived exosomes to mitigate the progression of colitis and inhibit CRC progression (Wang et al., 2022).

However, a number of studies have also shown that in tumour tissues, MSC-derived exosomes regulate tumour cells by delivering unique miRNAs to neighbouring cells and promote proliferation, migration, and invasion of tumour cells (Sharma, 2018). Recently, Zhang et al. (Zhang et al., 2021a) showed that BMSC-derived exosomes promote CRC cell proliferation and enhances CRC cell migration and invasion through miR-424, while the inhibition of miR-424 and elevation of TGFBR3 repress CRC cell proliferation and induce their apoptosis. Notably, they also found that miR-424 and TGFBR3 expression correlated with the degree of tumour differentiation, depth of tumour infiltration, TNM stage, vascular infiltration, lymphatic node metastasis (LNM), and distant metastasis in CRC patients. Studies also report that colon cancer stem cells (CCSCs) play an important role in cancer recurrence and chemotherapy resistance (Ricci-Vitiani et al., 2008; Gupta et al., 2019; Shiokawa et al., 2020). It is known that BMSC-derived exosomes increase the number of CSCs through the delivery of miR-142-3p, promoting a stem cell-like phenotype in CRC cells and thus worsening the progression of the disease (Li and Li, 2018). Several other studies further confirm the key role of MSC-derived exosomes in cancer progression by carrying microRNAs (miRNAs) and other types of molecules (Xu et al., 2019b; Jing et al., 2020). To this effect, a variety of miRNAs in MSC-derived exosomes could be potential therapeutic targets and their modulation may provide a new strategy for the treatment of CRC.

### Liver cancer

Liver cancer remains one of the most common and deadly malignancies worldwide, and its incidence is increasing every year (Llovet et al., 2021). It is estimated that more than 1 million people will die from liver cancer by 2030 (Villanueva, 2019). Globally, hepatocellular carcinoma (HCC) is the leading type of liver cancer, accounting for approximately 75% of the total incidence (Petrick et al., 2020). HCC mostly occurs in patients with cirrhosis (Forner et al., 2018), and the incidence of HCC is declining in some high prevalence areas, but increasing in many low prevalence areas (McGlynn et al., 2021). There are many clinical strategies for the treatment of HCC, including hepatic resection, liver transplantation, and transarterial chemoembolisation (TACE) (Liu et al., 2015). Among them, chemotherapy and immunotherapy are the better treatment options (Anwanwan et al., 2020). It is documented that MSC-derived exosomes have tissue repair effects, while drug-loaded exosomes have the potential to be used in the clinical treatment of liver cancer (Borrelli et al., 2018). As the specific mechanisms of MSC-derived exosome action in tissue repair and cancer continue to be explored, exosome-based therapies will become an alternative option for the clinical management of liver cancer.

Exosomes can promote liver regeneration, modulate inflammation and fibrosis, or inhibit tumour growth and metastasis in specific ways, and hold a promise as a novel therapeutic tool in a variety of liver diseases, including viral-associated liver disease, alcoholic liver disease (ALD), non-alcoholic fatty liver disease (NAFLD), and liver cancer (Borrelli et al., 2018; Balaphas et al., 2019; He et al., 2019; Ding et al., 2021b). The use of exosomes to control the progression of liver disease has therefore received increasing attention. Glutathione peroxidase 1 (GPX1) is an important antioxidant in the human body and is widely present in all cells (Deponte, 2013). GPX1 is closely associated with tumourigenesis and disease progression (Brigelius-Flohe and Kipp, 2009)and can be used as a biomarker for the clinical diagnosis and prognosis of certain malignancies (Cheng et al., 2019; Wei et al., 2020). Yan et al. (Yan et al., 2017)found that hucMSC-exosome promotes recovery from oxidative liver injury through the delivery of GPX1. In a mouse model of CCL4-induced liver injury, hucMSC-exosome reduced serum MDA, proinflammatory cytokine secretion, hepatic 8-OHdG expression, and apoptosis, exerting antioxidant and anti-apoptotic effects, thereby promoting liver injury repair and rescuing liver failure. Oxidative stress is often considered to be a key factor in the progression of chronic liver disease and hepatocarcinogenesis (Yu et al., 2014; Takaki and Yamamoto, 2015). Data from Jiang et al. (Jiang et al., 2018a) show that hucMSC-derived exosomes have a role in inhibiting liver tumour growth and that the specific mechanism of action is likely to be that hucMSC-Ex reduces oxidative stress in liver tumours and exhibits hepatoprotective functions through the antioxidant defense. Evidence has emerged that MSC-derived exosomes play an important tumour suppressive role in hepatocarcinogenesis *via* their molecular cargoes. For example, C5orf66-AS1 in MSC-derived exosomes enhances DUSP1 expression and inhibits ERK phosphorylation, thereby inhibiting HCC *in vivo* (Gu et al., 2021). This shows that MSC-derived exosomes and their cargoes have great potential for clinical applications in the treatment of liver disease and liver cancer.

MSC-derived exosomes have great potential as drug carriers with specific functions in combination with anti-cancer drugs for the treatment of HCC. Norethindrone (NCTD), a demethylated derivative of zebularine with enhanced anticancer activity and reduced toxicity of zebularine, has been used as a combination chemotherapeutic agent for liver cancer in clinical oncology treatment (Jiang et al., 2018b; Chi et al., 2019). NCTD is combined with 2-deoxy-glucose to inhibit the proliferation and migration of HCC cells. Currently, combining NCTD with biomaterials and anti-cancer drugs can greatly enhance the therapeutic effect of various HCCs and is a promising strategy (Li et al., 2021c; Gao et al., 2021; Yan et al., 2022). Liang et al. (Liang et al., 2021b)combined NCTD with purified BMSCs-derived exosomes by electroporation and found that the BMSC-Exos-NCTD drug delivery system could provide a sustained and slow drug release process that could effectively promote cell uptake, induce cell cycle arrest, reduce tumour cell proliferation, and increase apoptosis. The BMSC-Exos-NCTD exhibited stronger *in vivo* antitumour effects than NCTD alone, and the BMSC-derived exosomes not only function as good drug carriers but also inhibit the growth of HCC cells, acting as a dual therapeutic agent in combination with NCTD drugs.

The acquisition of drug resistance is one of the main reasons for the current suboptimal clinical outcome of liver cancer (Bao and Wong, 2021). Therefore, there is an urgent need to identify new targets and develop new therapeutic approaches to improve chemosensitivity in liver cancer. Considering the critical role of miRNAs in the progression of liver cancer and the acquisition of multidrug resistance (Giordano and Columbano, 2013), miR-199a-3p shows a downregulated trend in almost all HCC tissues, and its downregulation is associated with poor prognosis (Hou et al., 2011). MicroRNA-199a-3p has been shown to regulate hepatocyte apoptosis and hepatocarcinogenesis (Callegari et al., 2018; Li et al., 2020), affecting the sensitivity of human HCC cells to adriamycin (Fornari et al., 2010). Lou et al. (Lou et al., 2020) reported that mir-199a-modified exosomes from AMSCs could effectively enhance the sensitivity of HCC cells to chemotherapeutic agents by targeting the mTOR signaling pathway. In addition, intravenous injection of AMSC-derived exosomal miRNA-199a was further found to diffuse into tumour tissue, effectively enhancing the sensitivity of HCC cells to chemotherapeutic agents. In a previously established subcutaneous tumour-loaded model, the injection of mir-122-modified AMSC-derived exosome (AMSC-Exo-122) also significantly improved the antitumour efficacy of chemotherapeutic agents in HCC (Lou et al., 2015). The export of specific miRNAs *via* MSC-derived exosomes represents a novel strategy to improve the sensitivity of HCC chemotherapy.

### Pancreatic cancer

Pancreatic cancer is one of the deadliest types of human cancer due to its often advanced stage of detection and widespread treatment resistance (Mizrahi et al., 2020). According to GLOBOCAN 2020, there were 495,773 new cases worldwide and 466,003 deaths from pancreatic cancer (Papadakos et al., 2022). Unlike other tumours, although the incidence of pancreatic cancer continues to rise, the survival rates have barely improved (Khalaf et al., 2021). The continued increase in incidence is due in large part to an aging global population and key risk factors such as smoking, obesity, diabetes, and alcohol consumption (Klein, 2021). Of these, smoking is most strongly associated with pancreatic ductal adenocarcinoma (PDAC) (Park et al., 2021). Pancreatic cancer is insidious, fast progressing, early metastatic, and highly invasive (Zhang et al., 2021b; Cao et al., 2021; Ho et al., 2021; Yang et al., 2021), and often many patients have developed the metastatic disease by the time they are diagnosed (Stathis and Moore, 2010). Surgical resection, one of the best treatment strategies for pancreatic cancer, is not suitable for the majority of patients and the prognosis is poor (Paulson et al., 2013). Chemotherapy becomes one of the alternative strategies in the clinical management of pancreatic cancer, but there is still a possibility of local or systemic recurrence (Bednar and Pasca di Magliano, 2020; Mallya et al., 2021). A variety of immunotherapeutic approaches, including immune checkpoint inhibitors, cancer vaccines, pericyte transfer, and combinations with other immunotherapeutic agents, have been evaluated in numerous clinical trials (Schizas et al., 2020). As a potentially new and important tool to deliver anti-cancer drugs, MSCs and their expressed exosomes have been shown great potential for the treatment of pancreatic cancer (Loebinger and Janes, 2010).

MSC-derived exosomes can act as regulators of pancreatic cancer cell differentiation, proliferation, and apoptosis through inclusions such as miRNAs, mRNA, and proteins. miRNA-143 has been shown to function as a tumour suppressor in CRC (Zhang et al., 2012; Dougherty et al., 2021), breast cancer (Du et al., 2020)^,^ and ovarian cancer (Shi et al., 2018). In pancreatic cancer, the inhibitory effect of miRNA-143 has also been reported. Xu et al. (Xu et al., 2018) found that miRNA-143 expression was significantly reduced in pancreatic cancer tissues and could regulate the pancreatic cancer process by inhibiting its migration and invasion and promoting apoptosis. miR-143 could also be used to inhibit cancer by reducing the stability and expression of COX-2 mRNA in pancreatic cancer cells (Pham et al., 2013). A recent study found that miR-143-3p was significantly more expressed in MSC-derived exosomes than in exosomes from a human pancreatic cancer cell line (CFPAC-1) and could regulate KRAS, which is a key regulator and predictor of pancreatic cancer (Buscail et al., 2020; Wang et al., 2021b; Luo, 2021)and synergistically promote apoptosis and inhibit cell growth, invasion, and migration. In addition to miRNA-143, miRNA-124 has attracted increasing attention and research in pancreatic cancer, as its downregulation is significantly associated with poor prognosis in PDAC patients (Sun et al., 2016). Recently, a study conducted by Xu et al. (Xu et al., 2020) reports that miRNA-124 carried by BMSC-derived exosomes inhibits the proliferation, invasion, migration, and EMT of pancreatic tumour cells and induces apoptosis through the regulation of EZH2. *In vivo* and *in vitro* experiments have also demonstrated that miRNA-124 carried by exosomes enhances the sensitivity of pancreatic cancer cells to chemotherapy. In addition, miRNA-1231 (Shang et al., 2019) and miRNA-128-3p (Xie et al., 2022) in MSC-derived exosomes possess inhibitory effects on pancreatic cancer. In contrast, Ding and others showed that hucMSC-derived exosomes promote the growth of pancreatic ductal adenocarcinoma by transferring miR-100-5p, and that this promotion may be mediated by transferring miRNAs into PDAC cells to activate relevant signalling pathways (Ding et al., 2021a). In summary, MSC-derived exosomes could be considered a potential therapeutic vector for the treatment of pancreatic cancer.

Exosomes serve as an attractive nanoscale drug delivery platform for the treatment of pancreatic cancer as first demonstrated by Ding et al. (Ding et al., 2019), who used exosomes from hucMSCS to deliver exogenous miR-145-5p, which inhibited the proliferation and invasion of PDAC cells and increased apoptosis and cell cycle arrest while decreasing Smad3 expression. Pascucci et al. (Pascucci et al., 2014) merged paclitaxel with MSCs and released exosomes, which were found to significantly inhibit tumour growth *in vitro*, demonstrating for the first time that MSCs are capable of encapsulating and delivering active drugs *via* their secreted exosomes. Subsequently, various drug delivery platforms based on MSC-derived exosomes were devised. According to Zhou et al. (Zhou et al., 2020), by combining paclitaxel (PTX) and gemcitabine monophosphate (GEMP) with purified BMSC-derived exosomes to exploit the natural PDAC selectivity, they were able to construct a novel system for exosomal drug delivery with efficient targeting and penetration capabilities while bypassing the tumour ECM barrier. Similarly, Zhou et al. (Zhou et al., 2021) demonstrated an exosome-based dual delivery biological system was constructed from BMSC-derived exosomes, electroporation-loaded galectin-9 siRNA, and superficially modified with oxaliplatin (OXA). This drug delivery system further increases the accumulation of chemotherapeutic drugs in the tumour area while reducing systemic distribution, avoiding to a greater extent their side effects and effectively enhancing innate and adaptive anti-PADC immunity. Exosomes designed with targeted ligand modifications or genetic engineering still maintain their original properties and deliver chemotherapeutic drugs to tumour cells with greater efficiency, effectively enhancing tumour targeting and having great clinical application (Tian et al., 2014; Wang et al., 2017).

### Other GI cancers

Esophageal cancer (EC) is a lethal malignancy with a poor prognosis and is the sixth leading cause of cancer-related death worldwide (Smyth et al., 2017). Clinical treatment is mainly based on surgery. Due to the lack of effective early detection methods, EC patients are often diagnosed at an advanced stage with limited therapeutic intervention and high mortality. A number of related studies have shown that exosomes play an important role in EC progression, microenvironment remodeling, treatment resistance, and immunosuppression (Jing et al., 2021). Li et al. (Li et al., 2022b) reported that exosomal miR-200a could mediate the expression of Keap1 to promote the proliferation, migration, and invasion of esophageal cells, and inhibit apoptosis. In contrast, studies have also shown that exosomes play an inhibitory role in the occurrence and development of EC. It is documented that exosomal long non-coding RNA UCA1, a promising biomarker for esophageal cancer, exerts anticancer effects by inhibiting cell proliferation, invasion and migration, and colony formation in EC cells (Zhu et al., 2020b). The role of MSC-derived exosomes in EC is also worthy of further investigation. A recent study showed that BMSC-derived exosomal miR-19b-3p promoted EC cell proliferation, migration, invasion, and EMT by targeting SOCS1 and induced apoptosis (Deng et al., 2021).

Gallbladder cancer (GBC) is the most common malignancy of the biliary tract and is usually diagnosed late in the course of the disease, with a poor overall prognosis (Baiu and Visser, 2018). Clinical treatment is still predominantly surgical, but newer therapeutic modalities, such as molecular targeted therapy, have been used to improve the prognosis of patients with advanced disease (Kam et al., 2021; Okumura et al., 2021). Recent studies have shown that a variety of exosomal miRNAs, lncRNAs, miRNAs, and circRNAs can be used as clinical diagnostic biomarkers for GBC (Xue et al., 2020; Ren et al., 2021; Ueta et al., 2021). These include miR-182, which has been found to be associated with a wide range of cancers including breast, nasopharyngeal, prostate, and glioma (Liu et al., 2020b; Bai et al., 2021; He et al., 2021; Ma et al., 2022). Zheng et al. (Zheng et al., 2020)found that exosomal miR-182 significantly promoted the migration and invasion of GBC cells by inhibiting reversion-inducing-cysteine-rich protein with kazal motifs (RECK). Another study also documented that leptin enriched in GBC cell-derived exosomes could promote cell invasion and migration by regulating STAT3-mediated polarisation of M2 macrophages (Zhao et al., 2022). These observations show the association between exosomes and the development and progression of GBC, thus, the role of MSC-derived exosomes in this regard deserves to be further investigated and explored.

## Conclusion

The data presented in this study indicate that MSCs-derived exosomes play a crucial role in the development of GI cancers and have great potential in cancer therapy. A similar recent review by Zhao et al. (Zhao et al., 2021) which analyzed the effects of different MSC-derived exosomes on the development of gastrointestinal malignant tumours, made much the same deductions and pointed out future research directions to include the detailed exploration of the mechanisms by which MSC-derived exosomes regulate the development of GI cancers. The authors elaborated on the multiple effects of MSC-derived exosomes on most cancers and supplemented the discussion with detailed research progress of MSC-derived exosomes on various GI cancers, including less studied ones such as EC and GBC. Thus, it could be concluded that MSC-derived exosomes, as a cell-free substance, can exert inherent beneficial therapeutic effects, providing new therapeutic ideas for their application in cancer therapy, particularly for GI cancers.

Current challenges include large-scale culture and isolation techniques, optimal methods for long-term preservation of exosomes, rapid isolation, purification, quantification, and identification of exosomes. Therefore, in addition to focusing on the mechanism of the effect of MSC-derived exosomes on GI cancers, future research can also focus on other emerging research directions such asmass production of exosomes and preparation of engineered exosomes. Moreover, despite the many advantages of exosome research, there are some obstacles in their translation to clinical applications such as low targeting efficiency, safety, and susceptibility to phagocytosis by the immune system (Weng et al., 2021). Some emerging technologies also deserve attention, such as bottom-up extracellular vesicle assembly with precise control of lipid, protein, and RNA composition, which may stimulate the development of next-generation improved MSCs-exosomes (Staufer et al., 2021; Ferreira et al., 2022). Therefore, future research could focus on these research directions to develop more effective clinical applications.
